# Supplementary material for: Mature sperm small-RNA profile in the sparrow: implications for transgenerational effects of age on fitness
Source: Environ Epigenet. 2019 May 21;5(2):dvz007. doi: 10.1093/eep/dvz007 (PMC6527922; doi:10.1093/eep/dvz007)
Supplement: dvz007_Supplementary_Data [file dvz007_supplementary_data.zip › Matsushima et al Suppl Material final.docx]

Supplementary Material

Mature sperm small RNA profile in the sparrow:  implications for transgenerational effects of age on fitness

Mature sperm small RNA profile in the sparrow:  implications for transgenerational effects of age on fitness

Wayo Matsushima#1,2,3, Kristiana Brink#4, Julia Schroeder*4, Eric Miska*1,2,3, Katharina Gapp1,2

1 Gurdon Institute, University of Cambridge, Tennis Court Rd, Cambridge, CB2 1QN, UK.

2 Wellcome Trust Sanger Institute, Wellcome Genome Campus, Hinxton, CB10 1SA, UK.

3 Department of Genetics, University of Cambridge, Downing Street, Cambridge, CB2 3EH, UK.

4 Department of Life Sciences, Imperial College London, Silwood Park Campus, Ascot, United Kingdom

* corresponding authors, # these authors contributed equally to this work.

Supplementary Figure 1A

Exemplary electropherogram of sperm RNA isolated from sparrows as obtained by the Bioanalyzer small RNA Kit.

Supplementary Figure 1B

Electropherograms of sperm RNA isolated from aged and sparrows in their prime as obtained by the Bioanalyzer Pico 6000 Kit.

Y-axis: 200 nucleotides (nt) correspond to 28 seconds (s).

Supplementary Figure 2

Size distribution of small RNA next generation sequencing reads from prime and aged sparrow sperm. Error bars represent standard deviation (prime n= 8, aged n=9).
